# From Parts to Whole: A Systems Biology Approach to Decoding Milk Fever

**DOI:** 10.3390/vetsci12040347

**Published:** 2025-04-09

**Authors:** Burim N. Ametaj

**Affiliations:** Department of Agricultural, Food and Nutritional Science, University of Alberta, Edmonton, AB T6G 2P5, Canada; burim.ametaj@ualberta.ca

**Keywords:** milk fever, periparturient hypocalcemia, calcium homeostasis, dairy cows, reductionism, systems biology

## Abstract

Milk fever, or periparturient hypocalcemia, in dairy cows has traditionally been addressed as an acute calcium deficiency, leading to interventions like supplementation and adjustments in dietary cation–anion balance. Although these measures have improved clinical outcomes, milk fever remains a widespread and economically significant issue for the dairy industry. Emerging findings demonstrate that a narrow emphasis on blood calcium concentration overlooks the complex interactions of immune, endocrine, and metabolic pathways. Inflammatory mediators and bacterial endotoxins can compromise hormone-driven calcium regulation and induce compensatory calcium sequestration, thereby worsening both clinical and subclinical hypocalcemia. Recent insights from systems biology illustrate that milk fever arises from nonlinear interactions among various physiological networks, rather than a single deficiency. Consequently, this review contends that a holistic strategy including integrating nutrition, immunology, microbiology, genetics, and endocrinology is vital for comprehensive management and prevention of milk fever. By embracing a multidisciplinary perspective, producers and veterinarians can develop more robust, customized solutions that not only safeguard animal well-being but also bolster profitability. Such an approach promises to meet the evolving demands of modern dairy operations by reducing disease prevalence and enhancing overall productivity. Tackling milk fever through integrated methods may unlock possibilities for improved herd health and sustainable dairy farming.

## Simple Summary

Milk fever, also known as periparturient hypocalcemia, is a multifactorial condition that occurs in dairy cows around the time of calving, involving disturbances in calcium homeostasis and inflammatory responses. Traditionally, farmers and veterinarians deal with it by adding more calcium to the cow’s diet or changing certain minerals before calving. While this can help, milk fever still causes problems in many herds. Researchers have found that milk fever isn’t just about low blood calcium. A cow’s immune system, hormones, and metabolism all interact in ways that can affect calcium levels. For example, inflammation or bacterial endotoxins can upset normal hormone signals, causing the cow’s body to hold onto calcium instead of letting it circulate in the blood. This can exacerbate both clinical (obvious) and subclinical (hidden) cases of milk fever. Research using systems biology—a field that studies how all parts of the body work together—shows that milk fever arises from various “networks” in the cow’s body. These include nutrition, immunity, genetics, and more. By viewing milk fever as a multi-layered problem rather than a single nutrient issue, we can create more effective ways to prevent it. This broader approach could lead to healthier cows and better economic outcomes for dairy farms.

## 1. Introduction

Milk fever, also known as periparturient hypocalcemia, has been a persistent challenge in dairy herd management for over a century. Historically, research on milk fever primarily focused on its most prominent feature, acute reductions in blood calcium levels, leading to the development of interventions such as intravenous or subcutaneous calcium infusions and dietary cation–anion difference (DCAD) adjustments prior to parturition [1,2]. Early observations by veterinarians in the 19th and early 20th centuries [3,4,5,6] laid the groundwork for understanding milk fever as a disorder resulting predominantly from calcium deficiency. Pioneering studies by Fish [7,8] further established the association between hypocalcemia and clinical signs of milk fever, thereby driving the adoption of calcium-based treatment strategies that, while reducing mortality, did not completely resolve the incidence of the disorder [9].

Over time, accumulating evidence has highlighted the complexity of milk fever’s pathophysiology. Contemporary research indicates that a reductionist, calcium-centric model oversimplifies a condition that emerges from the complex relationships of metabolic, endocrine, immunological, and environmental factors. Mounting evidence suggests that subclinical inflammation, immune activation, and bacterial endotoxins have been implicated in altering hormone-mediated calcium homeostasis and contributing to adaptive calcium sequestration [10,11]. Furthermore, hypocalcemia is now recognized not as an isolated event but as a common thread in several other periparturient disorders—such as metritis, mastitis, ketosis, and displaced abomasum—through a self-reinforcing cycle of inflammation and immune dysfunction [12,13].

The objective of this article is to critically reassess the historical reductionist approach to milk fever and to advocate for a systems biology framework that embraces the multifactorial nature of the disorder. By integrating insights from genomics, proteomics, transcriptomics, and metabolomics, the aim is to provide a comprehensive understanding of the complex interactions that underlie milk fever [14,15]. This integrative perspective not only reveals previously unrecognized pathomechanisms but also paves the way for more robust diagnostic tools and targeted therapeutic interventions that address the full spectrum of factors influencing dairy cow health.

This review presents a novel perspective on the pathogenesis of milk fever, emphasizing its multifactorial nature beyond a simple calcium deficiency model. While emerging evidence supports key aspects of this framework, further research is needed to fully elucidate the mechanisms underlying the condition and to validate the proposed interactions.

In the sections that follow, we will review the evolution of milk fever research, detail the complex relationship of hypocalcemia with other periparturient diseases, and explore how emerging systems-level approaches can redefine our strategies for prevention and treatment. Ultimately, this manuscript seeks to shift the paradigm from a narrow focus on calcium restoration to a holistic view that considers the dynamic and interconnected nature of biological systems in dairy cows.

## 2. Reassessing Reductionism in Milk Fever Research

For more than a century, reductionist thinking has dominated the scientific study of milk fever (i.e., parturient paresis). Early 19th-century veterinarians [3,4] first noted that apparently healthy dairy cows could suddenly collapse near calving, yet their exploratory treatments often aimed at general inflammation or cerebral congestion rather than pinpointing any single biochemical issue [10]. By the early 20th century, as dairy cows were increasingly bred for high yields, empirical data began to confirm severe hypocalcemia in milk fever cows [5,7,8,9]. This emphasis on blood calcium initially appeared logical and fruitful: indeed, intravenous calcium therapy and dietary manipulations [1,2,16] reduced mortality and revealed key physiological principles.

Over time, however, it became evident that a one-dimensional focus overlooks the broader immunometabolic, endocrine, and environmental influences that converge during the periparturient period [11,17,18]. More recently, studies have shown that inflammatory responses frequently coincide with or precede hypocalcemia [13], strongly suggesting that low blood calcium is associated with immune activation rather than functioning as an isolated disorder. For instance, Martins et al. and Zhang et al. [13,19] reported that multiparous Holsteins with postpartum hypocalcemia had significantly higher concentrations of acute-phase proteins and proinflammatory cytokines, including serum amyloid A (SAA), haptoglobin, and tumor necrosis factor-α (TNF-α), associated with lower plasma calcium concentrations.

Serrenho et al. [20] similarly indicated that postpartum calcium supplementation slightly altered inflammatory markers in clinically healthy cows, emphasizing the tight relationships between calcium status and innate immunity. Meanwhile, research by [21,22,23] confirmed that lipopolysaccharide (LPS) challenges consistently induce pronounced inflammation and drop circulating calcium concentrations in lactating cows. Collectively, these findings suggest that targeting blood calcium alone cannot fully address the multifactorial nature of milk fever.

## 3. Hypocalcemia in Multiple Periparturient Diseases: The Common Role of Inflammation

**Figure 1:**
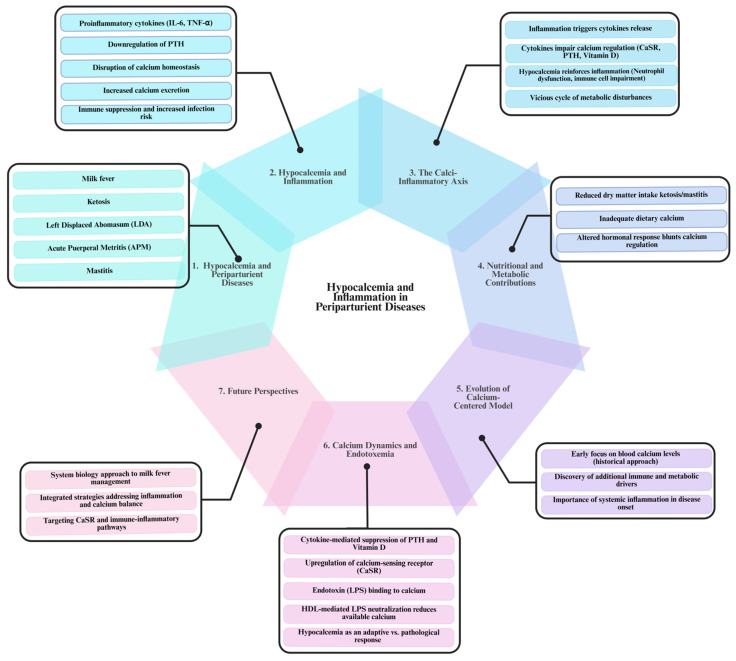
The bidirectional relationship between hypocalcemia and inflammation in periparturient diseases of dairy cows. The diagram illustrates the seven key aspects of the relationship between hypocalcemia and inflammation in periparturient diseases of dairy cows. (1) Hypocalcemia is a common feature of multiple periparturient disorders, including milk fever, ketosis, left displaced abomasum (LDA), acute puerperal metritis (APM), and mastitis, where persistently low serum calcium levels contribute to disease onset. (2) Inflammation plays a central role, as proinflammatory cytokines (IL-6, TNF-α) impair parathyroid hormone (PTH) secretion, disrupt calcium absorption, and increase calcium excretion, leading to immune suppression and increased infection risk. (3) The Calcium–Inflammation Axis creates a self-perpetuating cycle where inflammation disrupts calcium regulation, and hypocalcemia weakens immune responses, increasing disease susceptibility. (4) Nutritional factors, such as reduced dry matter intake in ketosis and mastitis, contribute to calcium imbalances by limiting dietary calcium availability. (5) Historically, milk fever research focused only on blood calcium levels, but current insights highlight the role of immune and metabolic factors in disease development. (6) Endotoxemia (LPS exposure) further disrupts calcium homeostasis by suppressing PTH, upregulating the calcium-sensing receptor (CaSR) and binding calcium, worsening hypocalcemia. (7) A systems biology approach is needed to integrate inflammation and calcium regulation into management strategies, moving beyond calcium supplementation alone to effectively prevent and treat periparturient diseases in dairy cows. [This diagram was created using BioRender.com in collaboration with Zohaib Saleem].

Multiple studies have provided data indicating that hypocalcemia (i.e., serum calcium concentrations < 2 mM [18]) is a ubiquitous feature not only of milk fever but also of several other periparturient disorders such as ketosis, left displaced abomasum (LDA), acute puerperal metritis (APM), and mastitis (Figure 1). For instance, Venjakob et al. [12] demonstrated that cows experiencing hypocalcemia around parturition exhibit persistently lower serum calcium levels on days 0, 1, and 3 postpartum—a pattern similarly observed in the aforementioned diseases. Additionally, cows with ketosis, particularly primiparous animals on days 3 and 7 postpartum, consistently show reduced calcium levels, and cows with LDA are several times more likely to be hypocalcemic [24,25,26]. Moreover, diminished serum calcium in cows with APM and mastitis suggests that hypocalcemia may both predispose animals to these disorders and arise from inflammatory processes.

The mechanisms driving the hypocalcemia–inflammation axis are complex and interrelated. Inflammatory responses triggered by conditions such as ketosis, LDA, APM, and mastitis lead to the release of proinflammatory cytokines, including interleukin-6 (IL-6) and TNF-α. These cytokines disrupt calcium homeostasis by downregulating parathyroid hormone (PTH) secretion and altering the function of the calcium-sensing receptor (CaSR), thereby reducing calcium absorption from the intestines, its mobilization from bones, and reabsorption in the kidneys, increasing its excretion [27]. As inflammation intensifies, the resulting hypocalcemia predisposes cows to further inflammatory challenges, reinforcing this self-escalating cycle.

Moreover, during inflammatory states, the activation of immune cells increases the demand for calcium to support essential cellular functions. Immunometabolomic studies [13,28] have shown that chronic low-grade inflammation sustains this heightened calcium requirement. Simultaneously, the ensuing hypocalcemia impairs critical immune functions, such as neutrophil activity [29], rendering cows more susceptible to infections and subsequent inflammatory responses. This reciprocal relationship exacerbates the metabolic disturbances already present, creating a self-perpetuating cycle in which hypocalcemia both results from and contributes to ongoing inflammation.

Nutritional factors further complicate this cycle. Disorders like ketosis and mastitis often reduce dry matter intake, leading to insufficient dietary calcium during periods of elevated metabolic demand [25,26]. This deepens the decline in serum calcium, undermining the animal’s ability to recover from inflammatory episodes and perpetuating the cycle of hypocalcemia and immune impairment. Inflammatory mediators also blunt normal hormonal mechanisms that help restore calcium homeostasis, allowing persistent hypocalcemia to exacerbate immune dysfunction and metabolic stress [27,29].

A critical aspect of this pathogenic loop is the bidirectional causality between hypocalcemia and inflammation (Figure 1). On one hand, low calcium impairs immune cell function, as evidenced by reduced neutrophil activity and lower immune cell counts [29,30,31], thereby increasing the risk of infections and further inflammatory responses. On the other hand, systemic inflammation—often induced by endotoxemia or infection—leads to the secretion of proinflammatory cytokines that impede calcium regulation via the CaSR [12,21]. Research by Zhang et al. [13] and Abuajamieh et al. [32] confirms that both metabolic disturbances and inflammatory responses are integral to the pathogenesis of milk fever. This reciprocal interaction creates a self-reinforcing loop: hypocalcemia-induced immune suppression increases the likelihood of infections or endotoxin translocation, which, in turn, triggers further inflammatory responses and disrupts calcium balance. Understanding the complex links between hypocalcemia and inflammation is crucial for developing integrated management strategies.

## 4. Moving Beyond a Narrow Focus on Calcium

From late gestation to the onset of lactation, dairy cows undergo profound physiological disturbances. While calcium homeostasis is undoubtedly crucial in milk fever pathophysiology [33,34], historical failures to control the disorder exclusively by restoring blood calcium illustrate the complexity at play [35,36]. Secondary drivers like immune activation, inflammatory signaling, and bacterial endotoxins can amplify a cow’s susceptibility [11]. Indeed, Zhang et al. and Zwierzchowski et al. [13,28] demonstrated that innate immune markers and metabolic disruptions can be detected weeks before the appearance of clinical milk fever. Therefore, strictly supplying calcium, whether via oral or intravenous routes, might stabilize acute hypocalcemia but neglect the multi-layered processes fueling onset and relapse [37,38].

### 4.1. Origins of the Calcium-Centered Model

#### Early Observations and Empirical Shifts

Although milk fever’s clinical profile was recognized by the mid-1800s [33,39], the focus on blood calcium started only in the early 20th century. Veterinarians like Schmidt [40] mistakenly attributed the disease to “viral toxins” in the udder, yet accidental discovery that udder inflation alone could rescue sick cows [41] emphasized the critical role of milk outflow. Soon after, researchers discovered that diseased cows had markedly lowered serum calcium near calving [34,42]. This finding triggered extensive research on PTH, vitamin D, and dietary calcium intake, leading to new prevention strategies such as DCAD manipulations [1,16,17]. Even so, milk fever continued to affect many herds, emphasizing that low calcium alone does not suffice to explain a disease shaped by multifactorial interactions [18].

## 5. Persistent Knowledge Gaps

Wide variability in a cow’s susceptibility—despite similar feeding regimens—reinforced that additional factors (genetic, epigenetic, immune reactivity, and bacterial endotoxins) can influence risk [11]. Historical data have shown that some cows used to experience repeated episodes of milk fever despite calcium supplementation [35,36]. Likewise, Zhang et al. [13] highlighted that proinflammatory cytokines and acute-phase proteins, including TNF-α, IL-6, SAA, and haptoglobin, are elevated well before the appearance of clinical signs of disease starting at 8 weeks prior to parturition and 10–11 weeks prior to disease occurrence. These inconsistencies revealed the limits of reductionist dogma around hypocalcemia and prompted renewed interest in inflammation, immunological responses, and metabolic networks during this critical periparturient window [38,43].

## 6. Calcium Dynamics During Inflammation and Endotoxin Exposure

As detailed in earlier sections, the bidirectional interaction between hypocalcemia and inflammation is a central driver of milk fever. In this section, we explore how inflammatory mediators, cellular adaptations, and endotoxin exposure modulate calcium homeostasis. The detailed mechanisms discussed herein highlight why a simple calcium supplementation strategy is insufficient to address all aspects of milk fever.

### 6.1. The Calci-Inflammatory Axis: Mechanisms and Emergent Nonlinear Dynamics in Calcium Homeostasis

Calcium homeostasis during inflammation involves processes far more complex than a mere dietary deficiency [44]. While insufficient calcium intake can exacerbate hypocalcemia, systemic inflammation, driven by proinflammatory cytokines and other mediators, fundamentally reshapes calcium mobilization and regulation in the body [44,45]. Hypocalcemia is frequently observed in multiple periparturient diseases of dairy cows, milk fever included, as well as in human patients during critical illness, sepsis, and severe burn injuries; however, the underlying mechanisms vary. Proinflammatory cytokines such as IL-1, IL-6, and TNF-α are often elevated in milk fever cows and critically ill patients [46,47] and have been shown to downregulate or alter the secretion and action of PTH [44,48] (Figure 1).

In parallel, the CaSR, the body’s “calciostat” in the parathyroid glands and kidneys, is upregulated in response to these inflammatory signals [44]. This upregulation lowers the set-point for extracellular calcium at which PTH and calcitonin are secreted. Consequently, even when serum calcium is low, the body may not respond adequately by increasing PTH secretion because the CaSR has “recalibrated” its target calcium concentration to a lower level. Simultaneously, calcitonin secretion is enhanced at these lower thresholds, which may promote calcium deposition in bone or other tissues. Such an inflammatory-driven resetting of calcium regulation helps explain why simple dietary calcium supplementation often fails to restore eucalcemia (i.e., serum calcium concentrations between 2.1–2.8 mM). Activating mutations of the CaSR can even intensify this hypocalcemic state, reflecting an intrinsic shift in the body’s “target” calcium concentration [44].

In conditions such as sepsis or endotoxemia, systemic elevations of cytokines (e.g., IL-1, IL-6) diminish PTH synthesis and impair active vitamin D production, collectively resulting in functional hypocalcemia that extends beyond mere insufficient dietary intake [44,49]. Additionally, biological systems rarely function via simple linear interactions; instead, they frequently exhibit a “butterfly behavior”, in which a single initiating event—such as inflammation—cascades through multiple regulatory pathways [50]. For instance, while inflammation may primarily disrupt calcium homeostasis through upregulation of CaSR, it also leads to amplified local cytokine production, altered hormonal secretion, and enhanced immune cell recruitment. Proinflammatory cytokines (e.g., IL-1 and IL-6) have been shown not only to suppress PTH secretion but also to further upregulate CaSR expression [44,47,51], effects that have been observed in critically ill patients, those with severe burns, and patients with rheumatoid arthritis [46,47,52,53,54]. Moreover, an increased concentration of extracellular calcium is often observed at sites of inflammation, where tissue injury or cellular damage releases calcium locally, functioning as a potent chemoattractant for immune cells [55]; thus, calcium serves not only as an indicator of tissue distress but also as an active secondary messenger.

Through its interaction with the CaSR, extracellular calcium has been demonstrated to enhance cytokine secretion and stimulate immune cell chemotaxis, thereby reinforcing the inflammatory response [56,57]. This dual functionality of the CaSR creates a feedback loop wherein inflammation and calcium regulation mutually reinforce one another. Initial inflammatory events induce cytokine release that upregulates CaSR expression [51,58], leading to systemic hypocalcemia and localized increases in extracellular calcium that further amplify immune responses [27,59]. The CaSR is highly expressed in the parathyroid glands, kidneys, and bones, as well as in diverse tissues such as the intestine, pancreatic islets, lungs, brain, skin, vasculature, and immune cells [60].

Clinical observations in humans further support this mutual relationship. For example, in patients with COVID-19, low serum calcium levels have been associated with increased disease severity and poor prognosis [61,62]. These findings suggest that inflammatory responses to viral infections may contribute to dysregulation of calcium homeostasis, which in turn exacerbates the inflammatory state—a concept reinforced by the ability of SARS-CoV-2 to perturb calcium dynamics [63,64].

In conclusion, the Calci-Inflammatory Axis is better seen as an intricate, dynamic network of interconnected pathways rather than a simple linear chain of cause and effect. Inflammation often triggers alterations in calcium homeostasis via CaSR upregulation, and the subsequent changes in extracellular calcium further amplify inflammatory responses. This bidirectional, nonlinear interplay—the “butterfly effect”—illustrates why interventions targeting only one aspect (e.g., calcium supplementation) are typically insufficient. Therapeutic strategies must therefore address both inflammatory signals and calcium regulatory pathways concurrently. Future research targeting the CaSR and its regulatory mechanisms, along with interventions that modulate both inflammation and calcium homeostasis, holds promise for breaking this axis and mitigating the pathological effects of chronic inflammation [65].

### 6.2. Calcium Sequestration and Cellular Adaptations

In inflammatory states, intracellular organelles in immune cells—including the endoplasmic reticulum (ER) and mitochondria—play a critical role in sequestering significant amounts of calcium as part of the immune response. This sequestration helps modulate cytokine release, oxidative bursts, and immune cell activation [66,67]. By buffering cytosolic calcium, these organelles help prevent excessive inflammatory signaling, thereby reducing potential tissue damage. However, this adaptation comes at a cost: it depletes extracellular ionized calcium, often manifesting as clinical hypocalcemia. Importantly, this hypocalcemia may represent an adaptive protective mechanism rather than a simple calcium deficiency [68,69]. As inflammation progresses, heightened activation of the CaSR can alter the body’s physiological “set point” for circulating calcium, making it difficult to restore normal calcium levels (eucalcemia) through supplementation alone.

A significant factor exacerbating this calcium imbalance is LPS. LPS can bind to free ionized calcium, forming LPS–calcium complexes that are transported to the liver for clearance by high-density lipoproteins (HDLs) [70,71]. Although this mechanism reduces LPS toxicity and helps protect against systemic inflammation [72,73], it can further contribute to transient hypocalcemia during severe infections or sepsis. Compounding this effect, proinflammatory cytokines—particularly interleukin-6 (IL-6)—can upregulate CaSR expression in the parathyroid glands and kidneys, leading to suppressed PTH and 1,25-dihydroxyvitamin D production [27,74]. As a result, multiple mechanisms—including cytokine-driven CaSR activation, intracellular calcium sequestration, and LPS-induced calcium binding—act synergistically to modulate the inflammatory response but reduce ionized calcium availability.

Although transient hypocalcemia can be beneficial by dampening the intensity of inflammation, prolonged or severe hypocalcemia poses significant risks. It can impair neuromuscular excitability, cardiac function, and overall physiological stability, worsening outcomes in critical illness [20,68,75]. Thus, maintaining a delicate balance is essential—harnessing the protective aspects of hypocalcemia to limit inflammation while avoiding its detrimental effects on essential physiological processes. Careful monitoring and management of calcium homeostasis are critical to achieving optimal immune function and promoting recovery during inflammatory conditions.

### 6.3. Endotoxin Translocation and Systemic Effects

Conditions such as subacute ruminal acidosis (SARA), mastitis, metritis, or hoof infections facilitate the translocation of LPS from the rumen, mammary gland, uterus, and hoofs [10,11,76]. Once in circulation, LPS binds to Toll-like receptor 4 (TLR4), triggering the release of proinflammatory cytokines (e.g., TNF-α, IL-1, and IL-6). This cytokine surge disrupts calcium balance by lowering bone mobilization, impeding renal calcium reabsorption, and inhibiting intestinal calcium absorption [23,77]. For instance, mastitis-induced inflammation increases both local and systemic cytokine concentrations, thereby distorting calcium-dependent cellular functions across tissues [11,78]. Additionally, uterine infections during the periparturient period can intensify these inflammatory loops, further aggravating calcium imbalances [79].

### 6.4. The Protective Role of HDL and Calcium Binding

High-density lipoprotein (HDL) plays a critical role in neutralizing LPS via calcium-mediated aggregate formation, which enhances the clearance of potentially harmful endotoxins [80,81,82]. Paradoxically, while this process is beneficial in clearing harmful endotoxins, it simultaneously reduces plasma calcium levels, leading to transient hypocalcemia. Traditionally, hypocalcemia has been viewed as a pathological condition or a sign of calcium deficiency. However, in this context, it may serve as an adaptive response aimed at diminishing LPS bioactivity and limiting inflammatory damage [80]. This paradox challenges the conventional understanding of hypocalcemia, suggesting that rather than being strictly pathological, it may represent a protective mechanism in response to endotoxin exposure. Emphasizing these integrative protective pathways is essential for distinguishing between functional, inflammation-driven hypocalcemia and true calcium deficiency.

The mechanisms described in this section reveal that inflammatory signals and endotoxin exposure intricately reprogram calcium homeostasis. Proinflammatory cytokines not only downregulate PTH secretion and alter CaSR sensitivity but also promote intracellular calcium sequestration and LPS binding, leading to a protective yet potentially maladaptive hypocalcemic response. These complex processes demonstrate how multiple regulatory pathways interact dynamically—a hallmark of emergent properties in biological systems. In essence, these findings reinforce that a simple focus on calcium supplementation is insufficient. Instead, a systems biology perspective is essential to integrate these diverse signaling networks and develop more effective, holistic management strategies for milk fever.

## 7. Consequences of a Single-Factor Focus

### 7.1. Oversimplification of Disease Complexity

Focusing on serum calcium alone, although it advanced early understanding of milk fever, can oversimplify the disease model [83]. Subclinical inflammation, insulin resistance, and microbial endotoxins have long been tied to parturient disorders [10,11,84]. Contemporary data reinforce that LPS translocation—whether from the rumen, uterus, infected hoofs, or mammary gland—may precipitate or exacerbate hypocalcemia [85,86,87]. Hence, prioritizing blood calcium might obscure equally critical factors in milk fever’s pathogenesis.

### 7.2. Practical Limitations and the Challenge of Reductionism in Complex Diseases

Traditional, one-dimensional approaches that focus solely on repleting blood calcium may temporarily stabilize acute symptoms but fail to resolve the underlying metabolic disturbances. Historical treatments such as udder inflation [40,41] demonstrate that interventions like halting colostrum flow can reduce visible signs in the short term, yet they do not correct deeper physiological dysfunctions. As a result, unresolved inflammation or latent infections may persist and reinitiate the disease process, contributing to milk fever as a chronic issue in dairy farming [12,13,88,89].

## 8. Why Reductionism Alone Struggles with Complex Diseases

### 8.1. The Challenge of Multi-Layered Interactions

While reductionist methods have provided valuable molecular insights, they are inherently limited when addressing multifactorial conditions like milk fever [90]. Early hypotheses relied on isolated concepts—such as “cerebral congestion”, “milk auto-intoxication”, or “parathyroid deficiency”—without fully considering the complex interplay among genetic factors, immune feedback loops, and environmental stressors [91,92,93]. A broader perspective reveals that cyclical inflammatory signaling and repeated endotoxin translocations emerge from an interconnected network of responses [94]. In this context, LPS-induced immune responses have been shown to exacerbate hypocalcemia, underscoring the integrative complexity of these disorders [69].

### 8.2. Limitations of Traditional Approaches in Understanding Milk Fever

Milk fever exemplifies the shortcomings of calcium-centric strategies. Although dietary supplementation and serum calcium monitoring can manage hypocalcemia, these interventions often neglect other critical factors—such as bacterial endotoxins, immunologic priming, proinflammatory cytokines, genetic predispositions, and subtle hormonal imbalances—that collectively disrupt calcium homeostasis [11,95]. The recurrence of milk fever highlights that such fragmented interventions address only a portion of the underlying pathology [76].

Recognizing the emergent properties inherent in complex diseases like milk fever underscores the necessity for a more integrative approach. By considering the full spectrum of interacting factors, we can develop more effective, long-lasting solutions that transcend the limitations of reductionist strategies.

## 9. Emergent Properties in Complex Diseases

### 9.1. Nonlinear Feedback and Interconnected Pathways

**Figure 2:**
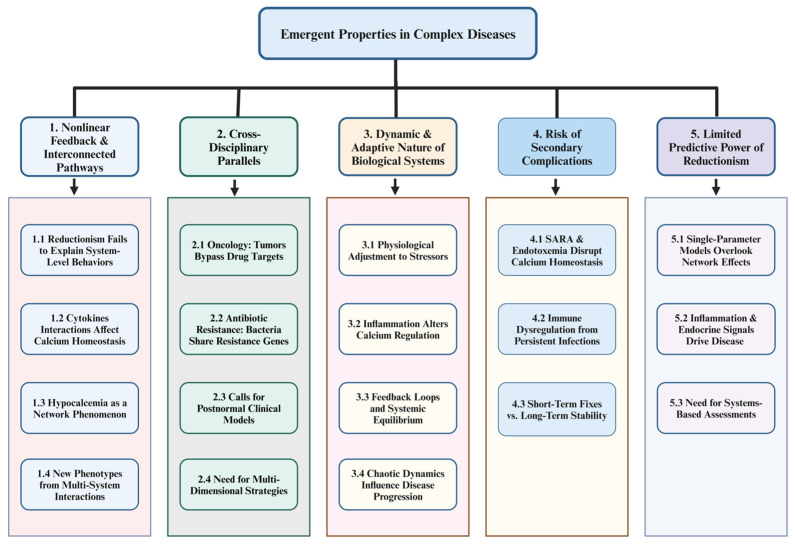
Emergent disease dynamics: a systems-based analysis of nonlinear feedback, adaptive physiology, and predictive limitations. The diagram represents the complex interplay of Nonlinear Feedback & Interconnected Pathways (Section 1) by showing how reductionist approaches fail to explain emergent system-level behaviors (1.1), how cytokines such as TNF-α and IL-6 nonlinearly affect calcium homeostasis (1.2), and why hypocalcemia is driven by broader network interactions rather than diet alone (1.3). Multi-system feedback loops can generate unexpected disease phenotypes (1.4). Cross-Disciplinary Parallels (Section 2) reveal similar principles: cancer cells circumvent targeted therapies by activating alternative pathways (2.1), bacteria disseminate resistance genes rapidly to undermine antibiotics (2.2), and “postnormal” clinical models emphasize the adaptive, network-based nature of disease (2.3), all reinforcing the necessity of multidimensional strategies for managing complex conditions (2.4). Highlighting the Dynamic and Adaptive Nature of Biological Systems (Section 3), organisms are shown to continually adjust to nutritional, environmental, and infectious stressors (3.1), with chronic inflammation reshaping calcium regulation through altered PTH and vitamin D pathways (3.2). These tightly coupled feedback loops maintain stability yet confound single-target treatments (3.3), and chaos theory provides insight into how disease progression patterns can become unpredictable (3.4). The Risk of Secondary Complications (Section 4) emphasizes that factors like subacute ruminal acidosis (SARA) and endotoxemia hinder calcium homeostasis (4.1), while persistent infections erode vital endocrine–immune interactions (4.2), illustrating why transient measures such as calcium boluses fail to achieve lasting equilibrium (4.3). Lastly, the Limited Predictive Power of Reductionism (Section 5) underlines that single-parameter models—focused solely on plasma calcium—overlook critical network-wide interactions (5.1) mediated by inflammatory and endocrine feedback loops (5.2). Consequently, a systems-based assessment is essential for accurately predicting disease trajectories and formulating robust preventative strategies (5.3). [This diagram was created using BioRender.com in collaboration with Zohaib Saleem].

A major limitation of reductionism is its inability to account for emergent properties—system-level behaviors arising from interactions among multiple subsystems [96,97]. During the periparturient period in dairy cows, heightened metabolic demands and chronic inflammatory states interact in nonlinear ways, leading to unpredictable outcomes. Calcium homeostasis is not governed by a single linear pathway but rather emerges from a dynamic interplay of immune, endocrine, and metabolic signals, where feedback loops can amplify or suppress key physiological processes depending on the inflammatory and metabolic context (Figure 2). Proinflammatory cytokines such as TNF-α and IL-6 can collectively impair bone mobilization, renal calcium reabsorption, and intestinal uptake. Consequently, hypocalcemia becomes less about a simple dietary deficiency and more about an emergent, network-driven phenomenon [98]. This “forest over the trees” perspective echoes the foundational insights of cybernetics and general systems theory, wherein biological organizations exhibit properties that surpass the sum of their parts [99,100]. When immune, endocrine, and metabolic signals interact, entirely new phenotypes may arise, requiring broader conceptual frameworks than can be provided by linear, cause-and-effect models [101,102].

### 9.2. Cross-Disciplinary Parallels

Such complexities are not confined to milk fever; they manifest throughout human and veterinary medicine. In oncology, for instance, a targeted therapy that blocks one signaling pathway often fails long-term because tumors avoid the blockage by activating alternative growth or survival pathways (Figure 2). Some breast cancers initially respond to estrogen receptor antagonists but eventually activate HER2 or PI3K/AKT pathways, bypassing their dependency on estrogen [103]. A parallel dynamic unfolds in antibiotic resistance: bacteria in diverse microbial communities can rapidly share resistance genes via plasmids or transposons, nullifying the efficacy of once-reliable treatments [104].

These examples underscore why reductionist models, which focus on individual molecules or pathogens, frequently overlook adaptive and compensatory mechanisms. Increasingly, calls for “postnormal” clinical models emphasize the adaptive, network-based features of disease, wherein subtle changes resonate throughout interconnected systems [105] (Figure 2). By targeting single factors, clinicians risk missing the broader web of redundancies and feedback loops that sustain pathological states. Consequently, multidimensional strategies—designed to address the entire network—offer a more robust path to controlling complex diseases [106,107].

### 9.3. Dynamic and Adaptive Nature of Biological Systems

#### 9.3.1. Continuous Physiological Adjustments

Living organisms continually adapt to nutritional, infectious, and environmental stressors, complicating single-factor interventions [108,109]. In the context of milk fever, administering calcium supplements may temporarily elevate serum calcium. However, persistent inflammation—such as that originating from mastitis or metritis—can suppress PTH responsiveness and alter vitamin D metabolism [45,110]. Even if plasma calcium is restored, the upregulation of the CaSR and disruption of hormonal feedback loops can keep the regulatory system maladjusted. This reverberates in the robust networks described by Kitano and others [111,112], where multiple feedback loops stabilize the organism yet complicate simplistic, one-dimensional treatments (Figure 2).

Such resilience is further shaped by factors like circadian rhythms, chaotic dynamics, and unanticipated feedback effects [113,114]. Hence, measures like calcium boluses alone may not suffice if the broader inflammatory milieu remains unaddressed. The cow’s recalibration of its calcium set-point—via the CaSR and modified PTH release—illustrates how interconnected physiological networks aim for equilibrium yet can generate emergent challenges for clinical management. As Lindberg noted historically [111], complex biological transformations rarely yield to purely mechanistic “fixes”, underscoring the necessity of integrative, adaptive strategies.

#### 9.3.2. Risk of Secondary Complications

Reductionist treatments also risk missing latent triggers that continuously undermine homeostasis. Subacute ruminal acidosis (SARA) compromises the integrity of the rumen wall, enabling low-level translocation of LPS and creating a background of persistent endotoxemia [115]. This chronic immune activation disrupts both endocrine signaling and calcium regulation, priming animals for recurrent bouts of hypocalcemia. Similarly, uterine infections can activate systemic inflammation that suppresses PTH secretion and disturbs vitamin D metabolism. These concealed triggers erode the animal’s capacity to maintain stable calcium levels, even when calcium boluses are administered.

Kitano’s perspectives on network resilience [101] highlight the pitfall of overlooking minor elements within biological systems. Simply correcting hypocalcemia offers a short-term fix but neglects factors like rumen integrity or low-grade inflammation. Over time, these unresolved issues reinitiate the metabolic cascade, undermining the predictive power of any single-parameter intervention [116]. Addressing milk fever sustainably thus demands a multi-layered approach: replenishing plasma calcium while managing inflammation, ensuring rumen health, and stabilizing the immune–endocrine axis.

### 9.4. Limited Predictive Power

Because immune, endocrine, and metabolic frameworks in dairy cows are interconnected, plasma calcium levels alone rarely capture the complexity of PTH action, vitamin D dynamics, bone mobilization, and inflammation [89,117]. A cow remaining at marginal calcium levels may still be predisposed to milk fever if her PTH secretion is inhibited by proinflammatory cytokines (Figure 2). Relying on a strict numeric threshold for “normal” calcium thus overlooks the subtle interactions of timing, tissue-specific responses, and contextual stressors—especially during the periparturient period.

For example, a herd experiencing subclinical mastitis may show only borderline-low calcium levels, yet the accompanying inflammatory environment could suppress PTH release and disrupt vitamin D metabolism. Over time, this interplay can tip multiple cows from borderline calcium status into outright hypocalcemia. Solely tracking plasma calcium misses how chronic inflammation, immune signaling, and endocrine factors converge to drive the clinical manifestation of milk fever. Such scenarios highlight the need for multifactorial, systems-based assessments that integrate diverse biomarkers and physiological feedback loops.

### 9.5. Moving Beyond Reductionism: Systems Biology and Integrative Approaches

Overcoming reductionist constraints in complex diseases like milk fever has led to growing interest in systems biology and integrative research models [118,119]. These approaches acknowledge that diseases emerge from multi-level interactions among genetic, metabolic, environmental, and immunological networks [108]. Drawing on principles from cybernetics [99] and general system theory [100], systems biology urges examining how various factors collectively produce system-wide behaviors—rather than isolating a single parameter like plasma calcium.

In a systems-based study of milk fever, researchers might track not only calcium metabolism but also hormonal fluctuations (PTH, calcitonin), inflammatory pathways, genotype, epigenetic influences, and nutrient fluxes [120]. For instance, subtle disruptions in rumen function due to SARA can lead to chronic, low-level endotoxin exposure, fueling persistent inflammation. Although administering a calcium bolus may temporarily normalize plasma calcium, cytokines such as IL-1 and IL-6 can sustain a cycle of suppressed PTH and disrupted vitamin D metabolism, thus perpetuating hypocalcemia [45,110]. This “butterfly effect” emphasizes how minor, localized triggers can spread through interrelated pathways, reprogramming the physiological set-point.

By adopting systems biology, researchers can detect hidden triggers of disease and develop computational models or high-throughput tools that target foundational imbalances, rather than merely alleviating signs of disease. This integrative view offers a pathway to more enduring, multi-layered therapies, ensuring that recurrent hypocalcemia is addressed at its roots. Ultimately, these broader strategies have the potential to transform milk fever management and improve overall herd health by accounting for the emergent and adaptive nature of biological systems.

## 10. Pioneering Systems Biology Approaches to Decoding the Century-Old Enigma of Milk Fever

Milk fever, traditionally recognized as a disorder of hypocalcemia occurring around calving in dairy cows, is now understood to be a complex metabolic and immunological condition. Recent advancements in genomic, proteomic, transcriptomic, and metabolomic technologies have started to deepen our understanding of milk fever, revealing that its etiopathology extends far beyond simple calcium deficiency. This section explores how these “omics” approaches throw light onto the complex interactions between calcium homeostasis, immune function, genetic factors, and metabolic alterations in the development of milk fever.

### 10.1. Genomic Contributions to Understanding Milk Fever

**Figure 3:**
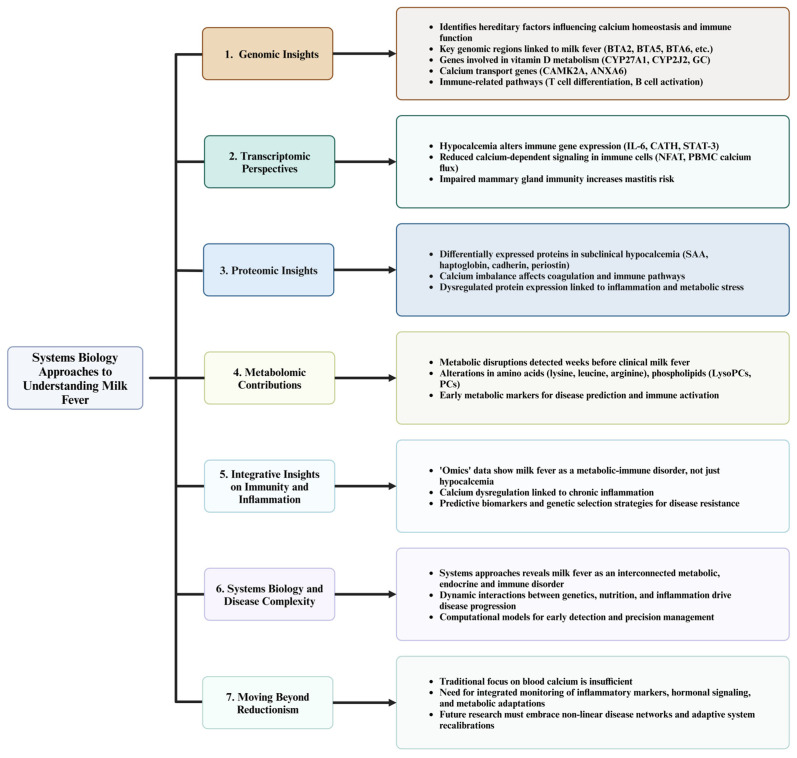
Systems biology approaches to decoding the complexity of milk fever in dairy cows. This diagram illustrates seven key aspects of systems biology approaches to understanding milk fever in dairy cows. (1) Genomic reports reveal key hereditary factors influencing calcium homeostasis and immune function, identifying candidate genes and pathways associated with milk fever susceptibility. (2) Transcriptomic studies show how hypocalcemia alters immune gene expression, reducing calcium-dependent signaling and increasing disease susceptibility, particularly in the mammary gland. (3) Proteomic insights highlight differentially expressed proteins in subclinical hypocalcemia, linking calcium imbalance to inflammation, coagulation disorders, and metabolic stress. (4) Metabolomic contributions identify metabolic disruptions weeks before clinical disease onset, with changes in amino acids, phospholipids, and inflammatory markers indicating early risk factors. (5) Integrative insights demonstrate that milk fever is not solely a calcium deficiency, but a metabolic-immune disorder influenced by chronic inflammation, suggesting predictive biomarkers and genetic selection strategies for disease resistance. (6) Systems biology approaches uncover the complexity of milk fever as an interconnected metabolic, endocrine, and immune disorder, emphasizing the importance of computational models for early detection and precision management. (7) Moving beyond reductionism, this framework highlights the need to shift from a sole focus on blood calcium levels to a broader, integrated monitoring of inflammatory markers, hormonal signaling, and metabolic adaptations for more effective prevention and management strategies. This diagram was created using BioRender.com in collaboration with Zohaib Saleem.

Genomic studies have identified the hereditary aspects of calcium regulation and immune function, revealing candidate genes and pathways implicated in milk fever susceptibility. In a study by Pacheco et al. [95], the authors are reporting key genomic regions and candidate genes associated with susceptibility to milk fever in Holstein dairy cows, revealing significant genetic mechanisms underlying this metabolic disorder. Whole-genome scanning pinpointed eight genomic regions across chromosomes BTA2, BTA3, BTA5, BTA6, BTA7, BTA14, BTA16, and BTA23, which collectively explained a considerable portion of the genetic variance linked to milk fever incidence. Particularly, five genes—*CYP27A1*, *CYP2J2*, *GC*, *SNAI2*, and *PIM1*—were implicated in vitamin D metabolism, transport, and signaling (Figure 3). Given that vitamin D is a critical regulator of calcium homeostasis, these findings highlight its role in the development of milk fever. Additionally, genes involved in calcium ion transport (*CAMK2A* and *ANXA6*) further underline calcium imbalance as a central component of this disorder. Gene-set enrichment analysis also revealed functional pathways related to immune response regulation (T cell differentiation and B cell activation), and protein phosphorylation. These enriched pathways provide molecular insights into how genetic factors influence immune responses and calcium dynamics during the periparturient period. Overall, the integration of gene mapping and biological pathway analysis offers new opportunities for improving milk fever resistance through marker-assisted selection and targeted breeding strategies.

Similarly, a study by Cavani et al. [121] reports how gene mapping and gene-set enrichment analyses provide valuable insights into postpartum hypocalcemia in Holstein cows. The authors indicated that whole-genome scans identified key genomic regions on BTA5, BTA6, and BTA16, harboring important genes such as *GC* (vitamin D binding protein), *LRP6* (bone remodeling), and *CACNA1S* (Ca ion channels), which are critical for calcium homeostasis and adaptation to the increased Ca demand during lactation. The genomic region on BTA16 also included *LRRC38* and *KCNK9*, which regulate potassium ion channels, affecting parathyroid hormone secretion and Ca regulation.

Additionally, the gene-set enrichment analysis from the same authors revealed pathways related to calcium ion binding, calcium signaling, and immune regulation, emphasizing the interconnected roles of Ca homeostasis and immune responses. The study found that poor Ca regulation during lactation can impair immune function, increasing susceptibility to periparturient diseases. Moreover, the analysis also identified significant immune-related pathways, suggesting that disruptions in Ca balance affect immune function during the periparturient period. Genes involved in protein kinase signaling, nucleotide binding, and inositol signaling were found to modulate cellular Ca signaling in immune responses. The study suggests that poor Ca regulation may contribute to immune suppression, which is consistent with the role of hypocalcemia in increasing the risk of diseases like retained placenta, metritis, and mastitis.

### 10.2. Transcriptomic Perspectives on Calcium and Immune Regulation

Transcriptomic analyses have provided valuable insights into immune dysregulation during milk fever. For instance, Ohtsuka et al. [122] demonstrated that cows with hypocalcemia after calving exhibited significantly lower expression of key immune-related components, especially IL-6 and cathelicidin (CATH), in milk somatic cells compared to healthy cows. Interleukin-6, a cytokine crucial for immune regulation and inflammatory responses, showed a significant decrease in the hypocalcemia group, especially at 4 weeks postpartum, suggesting impaired immune responses in the mammary gland (Figure 3). Cathelicidin, an antimicrobial peptide induced by IL-6 and essential for combating bacterial infections, also exhibited lower expression, although the difference was not statistically significant. The nuclear factor of activated T-cells (NFAT), which is calcium-dependent and critical for IL-6 regulation, remained stable and lower in the hypocalcemia group, indicating disrupted calcium-dependent signaling pathways. The STAT-3 transcription factor, involved in immune cell proliferation and survival, showed delayed activation in hypocalcemic cows, further supporting immune dysfunction. These findings highlight that hypocalcemia impairs calcium signaling and key immune mechanisms in the mammary gland, increasing susceptibility to infections such as mastitis during early lactation. The study emphasizes the role of calcium-dependent pathways in maintaining optimal immune defenses and the need for interventions to mitigate immune suppression in hypocalcemic cows.

In an earlier study, Kimura et al. [29] demonstrated that hypocalcemia during the periparturient period in dairy cows significantly impairs calcium signaling in peripheral blood mononuclear cells (PBMCs), contributing to immunosuppression. The increased calcium demand before and after parturition leads to a progressive depletion of intracellular calcium stores within the endoplasmic reticulum (ER) of PBMCs, starting several days before calving. Cows that developed milk fever showed a greater reduction in intracellular calcium stores and a blunted calcium flux response to immune activation compared to healthy cows. Restoration of blood calcium levels through intravenous calcium infusion improved PBMC calcium flux, suggesting that extracellular calcium status directly influences intracellular calcium dynamics and immune cell function. The study found significant correlations between plasma calcium, releasable calcium from ER stores, and the magnitude of calcium flux in PBMCs, confirming that systemic calcium stress precedes measurable hypocalcemia, particularly in cows developing milk fever. The impaired calcium signaling was associated with reduced PBMC activation, which compromises cytokine production and immune responses. These findings highlight that decreased intracellular calcium stores limit immune cell activation, making hypocalcemic cows more susceptible to infections during the periparturient period. The study highlights the interrelation between calcium and immunity and associated health complications in dairy cows.

### 10.3. Proteomic Insights into Pathophysiology

A proteomic study by Fan et al. [123] identified 398 differentially expressed plasma proteins in dairy cows with subclinical hypocalcemia (SH), with 24 proteins confirmed through iTRAQ/LC-MS/MS analysis. Two key Ca-associated proteins, cadherin and periostin, were upregulated in SH cows, suggesting a role in cell adhesion, protein binding, and potentially initiating mechanisms to stimulate blood Ca levels. Several immune-related proteins were also upregulated, including SAA and haptoglobin, which are critical in acute phase responses and indicate activation of innate immunity in SH cows. Additionally, differentially expressed proteins related to blood coagulation and complement pathways, such as serpins, platelet factors, and complement factors, were identified, showing that reduced blood Ca alters coagulation processes and weakens immune defenses. These disruptions can predispose cows to periparturient diseases, including mastitis, metritis, retained placenta, and ketosis. The findings highlight how Ca imbalance in SH affects key metabolic and immune pathways, emphasizing its significant impact on dairy cow health and productivity (Figure 3).

Another study by Wang et al. [124] found six key plasma proteins differentially expressed in dairy cows with SH, emphasizing their roles in calcium regulation, immune responses, and inflammation. Serum albumin and fibrinogen alpha chain were upregulated, with serum albumin playing a critical role in transporting calcium ions, hormones, and metabolites, and fibrinogen contributing to blood coagulation and inflammation. The amyloid beta A4 protein, known to regulate calcium signaling and potentially disrupt calcium homeostasis, was also upregulated, along with neurosecretory protein VGF, which is associated with inflammatory responses and nerve injury repair. On the other hand, apolipoprotein A-II and SAA proteins were downregulated, potentially affecting lipid metabolism and immune regulation via HDL. The differential expression of these proteins suggests that SH contributes to immune suppression, inflammation, and altered calcium transport, ultimately impairing dairy cow health and increasing susceptibility to diseases such as mastitis and metritis. These findings provide new insights into the molecular mechanisms of SH and the interaction of immunity and calcium regulation pathways during milk fever in dairy cows.

### 10.4. Metabolomics’ New Contributions That Expand the Understanding of Milk Fever

A recent study by Zwierzchowski et al. [28] provided a comprehensive metabolomic analysis of dairy cows affected by milk fever, emphasizing significant metabolic disruptions beginning several weeks before the clinical onset of the disease and persisting postpartum. Key findings include alterations in 31 serum metabolites that varied consistently across pre-milk fever, during the disease occurrence, and post-milk fever stages, primarily amino acids (AAs), lysophosphatidylcholines (LysoPCs), phosphatidylcholines (PCs), and acetylornithine [28]. Pre-milk fever cows showed increased concentrations of lysine, leucine, and isoleucine, immunopotent AAs associated with the synthesis of proinflammatory cytokines, acute-phase proteins, and antimicrobial peptides, supporting the immune response to potential endotoxemia [28]. Higher concentrations of arginine and citrulline were also observed, suggesting M1 macrophage activation and nitric oxide production, crucial for antimicrobial defense (Figure 3).

During the week of milk fever diagnosis, alterations in numerous AAs, biogenic amines, and PCs were detected, with the persistence of changes at +4 and +8 weeks postpartum [28]. Of note, increased plasma kynurenine levels in milk fever cows were linked to modulation of inflammation and endotoxin tolerance. Phatidylcholines and LysoPCs showed increased concentrations, reflecting both the immune response and its control, as LysoPCs are known activators of immune cells and modulators of proinflammatory responses [28].

Metabolic pathway analyses revealed that milk fever primarily disrupted amino acid, purine, pyrimidine, and aminoacyl-tRNA metabolism, highlighting the role of these pathways in immune regulation and metabolic homeostasis. Increased acetylornithine indicated overactivation of glutamine catabolism and ornithine production, which is essential for polyamine synthesis and immune modulation. This comprehensive metabolic shift emphasizes the complexity of milk fever, demonstrating that beyond hypocalcemia, the condition involves extensive metabolic dysregulation.

### 10.5. Integrative Insights: Inflammation and Immunity in Milk Fever

Mounting evidence from genomic, transcriptomic, proteomic, and metabolomic investigations suggests that milk fever is far more complex than a mere deficit in calcium homeostasis. Rather, it emerges as a complex metabolic syndrome interrelated with chronic inflammation and immune dysregulation. Genomic analyses have revealed candidate genes and pathways that not only regulate calcium transport but also critically modulate immune functions. For instance, studies have identified key genomic regions on multiple bovine chromosomes harboring candidate genes such as *CYP27A1*, *CYP2J2*, *GC*, *SNAI2*, and *PIM1*, which are involved in vitamin D metabolism, calcium ion signaling, and immune activation [95,121]. In addition, genes such as *CAMK2A* and *ANXA6* highlight the role of calcium transport in the onset of milk fever, further emphasizing the dual impact on metabolic and immunological processes (Figure 3).

Transcriptomic research provides additional mechanistic insights into the immune suppression associated with hypocalcemia. Alterations in the expression of key cytokines (e.g., IL-6) and antimicrobial peptides (e.g., CATH) in milk somatic cells indicate that the mammary defense system is compromised in hypocalcemic cows, increasing their susceptibility to mastitis and other periparturient infections [124]. Furthermore, studies have documented blunted calcium flux in PBMCs, which compromises immune cell activation and cytokine production, a clear demonstration that calcium-dependent signaling pathways are essential for maintaining effective immune responses during the critical periparturient period [29].

Proteomic investigations lend further support to this integrative perspective. Differential expression of proteins implicated in acute phase responses, coagulation, and inflammation, such as SAA, haptoglobin, fibrinogen, cadherin, and periostin, has been observed in periparturient cows exhibiting subclinical hypocalcemia or early-stage milk fever [122,123]. These findings suggest that proteomic disruptions not only affect calcium regulatory mechanisms but also contribute to a persistent inflammatory state that compromises overall health.

Metabolomic perspectives further expand this integrative view by revealing chronic inflammatory processes and early biochemical perturbations that precede clinically detectable milk fever. Elevated levels of specific AAs, PCs, LysoPCs, and other immune-regulatory metabolites [28] highlight a state of metabolic reprogramming. These alterations not only reflect a heightened inflammatory status but also suggest potential biomarkers for early risk assessment of milk fever.

Collectively, these converging “omics” data illustrate that milk fever is an emergent disorder characterized by interdependent genetic, metabolic, immunological, and endocrine alterations. This systems-level perspective improves our understanding of milk fever pathophysiology, emphasizing that disruptions in calcium homeostasis are intimately linked with chronic inflammation and immune responses. By identifying critical risk biomarkers and elucidating the interconnected pathways that contribute to hypocalcemia and immune dysfunction, these integrative insights pave the way for holistic prevention strategies. Ultimately, such strategies may include the development of predictive models for early disease detection, precision nutritional interventions, and targeted genetic selection programs aimed at enhancing resilience and ensuring the sustainable health of modern dairy herds.

The study of milk fever has traditionally been rooted in a reductionist framework, wherein individual components, such as plasma calcium levels, PTH secretion, or vitamin D metabolism, were studied and analyzed to explain the disease’s clinical manifestations. This classical approach, although instrumental in establishing early therapeutic strategies (e.g., calcium supplementation and dietary adjustments), inherently simplifies a complex, multifactorial disorder. Reductionism seeks to deconstruct biological phenomena into discrete, manageable parts. In the case of milk fever, this approach led to a predominant focus on correcting hypocalcemia as if it were the sole cause, while insufficiently considering the interrelated roles of inflammatory mediators, endocrine signaling, and metabolic adaptations.

## 11. Discussion

The study of milk fever has traditionally been rooted in a reductionist framework, wherein individual components, such as plasma calcium levels, PTH secretion, or vitamin D metabolism, were studied and analyzed to explain the disease’s clinical manifestations. This classical approach, although instrumental in establishing early therapeutic strategies (e.g., calcium supplementation and dietary adjustments), inherently simplifies a complex, multifactorial disorder. Reductionism seeks to deconstruct biological phenomena into discrete, manageable parts. In the case of milk fever, this approach led to a predominant focus on correcting hypocalcemia as if it were the sole cause, while insufficiently considering the interrelated roles of inflammatory mediators, endocrine signaling, and metabolic adaptations.

However, as our understanding deepens, the limitations of a reductionist view become increasingly apparent. Milk fever is not merely a consequence of a singular electrolyte imbalance but rather the endpoint of dynamic interactions among multiple physiological systems. Latent triggers, such as SARA, uterine infections, mastitis, hoof infections, or low-level LPS translocation, can quietly destabilize homeostatic loops, leading to recurrent hypocalcemia even when plasma calcium appears near normal [115]. Lindberg [111] has long noted that the inherent complexity of biological systems rarely yields to simple, mechanistic fixes. In effect, when underlying inflammatory processes persist, they reset endocrine feedback loops, via mechanisms like CaSR upregulation, that continuously undermine corrective measures.

A compelling demonstration of this concept comes from the experimental work of Goff et al. [125], who provided critical support for the Calci-Inflammatory Axis hypothesis. Their study utilized a mastectomy model in which cows were surgically deprived of their mammary glands prior to calving. These cows, despite undergoing the hormonal cascade of parturition, did not develop hypocalcemia. This striking finding illustrates that removal of the udder not only eliminates the calcium sink associated with colostrogenesis but also removes a potential source of inflammatory stimuli—particularly lipopolysaccharides (LPS) derived from Gram-negative bacteria often implicated in subclinical mastitis. It could be that the absence of hypocalcemia in these cows reflects, at least in part, the elimination of mammary-derived LPS and other inflammatory mediators, thus supporting the view that systemic inflammation, potentially triggered by microbial translocation from a compromised mammary gland, plays a central role in the disruption of calcium homeostasis. Thus, this study provides a mechanistic link between mammary inflammation and the calcium dysregulation observed during the periparturient period, emphasizing inflammation as a fundamental driver of early lactation metabolic disturbances.

In contrast, systems biology represents a paradigm shift that acknowledges the multi-level interactions governing complex diseases such as milk fever. This approach integrates insights from genomics, transcriptomics, proteomics, and metabolomics, providing a holistic view of how genetic, metabolic, environmental, and immunological networks converge to drive disease pathology [107]. Systems biology is deeply rooted in the principles of cybernetics [99] and general system theory [100], both of which emphasize understanding the “forest” as well as the “trees”. By considering the network as a whole, researchers can identify emergent properties, such as the “Calci-Inflammatory Axis”, that arise from nonlinear feedback loops and adaptive recalibrations within the system.

This systems-based view is further supported by recent research from McArt and colleagues [126,127], who advanced our understanding of subclinical hypocalcemia (SCH) by classifying it into three distinct temporal phenotypes: transient (tSCH), persistent (pSCH), and delayed (dSCH). These plasma calcium dynamics, captured from day 1 to day 4 postpartum, reflect deeper physiological and immunological processes. Cows experiencing tSCH—characterized by a brief drop in calcium followed by rapid normalization—showed superior dry matter intake, higher milk production, and reduced disease incidence compared to their dyscalcemic counterparts. McArt et al. interpret this transient hypocalcemia as a hallmark of homeorrhesis, a systemic metabolic realignment that prioritizes milk synthesis through tightly regulated calcium allocation. In this view, tSCH represents a beneficial immunometabolic recalibration, potentially facilitating the resolution of inflammation. In contrast, cows with pSCH or dSCH display prolonged or delayed calcium recovery, elevated inflammatory biomarkers, and greater disease susceptibility—traits indicative of a failure to restore metabolic balance. These findings align conceptually with the Calci-Inflammatory Axis hypothesis by suggesting that calcium oscillations may serve as reflections not only of mineral metabolism but also of systemic inflammatory status and resilience. While recent work implicating LPS and other inflammatory triggers has provided intriguing correlational data, we acknowledge that conclusive evidence for a novel mechanistic pathway remains to be fully elucidated.

Further reinforcing this perspective, Horst et al. [38] critically challenged the traditional metabolic dogmas of transition cow health and proposed that hypocalcemia, along with elevations in NEFA and ketones, is not causative of disease but rather a downstream consequence of immune activation. According to their findings, systemic inflammation—originating from microbial triggers in the mammary gland, uterus, or gastrointestinal tract—precedes the onset of hypocalcemia by inducing anorexia, disrupting nutrient partitioning, and redirecting metabolic resources toward the immune response. Inflammatory cytokines generated under these conditions suppress PTH secretion and interfere with vitamin D metabolism, thereby impairing calcium mobilization and absorption. This reconceptualization powerfully aligns with the Calci-Inflammatory Axis hypothesis by reframing hypocalcemia not as a primary defect of mineral balance but as a biomarker of inflammation-induced endocrine and metabolic dysfunction. Their work lends further weight to the systems biology approach by emphasizing the importance of addressing the immune–metabolic interface as a means to understand and manage milk fever more effectively.

Overall, a systems-based investigation into milk fever does not merely monitor plasma calcium; it also tracks hormonal signaling (including PTH and calcitonin dynamics), inflammatory cascades, nutrient fluxes, and even epigenetic modifications [120]. Consider a dairy cow that experiences hypocalcemia. A reductionist intervention might focus on administering a calcium bolus, temporarily restoring plasma calcium. However, if low-level endotoxin exposure persists and drives a chronic inflammatory state, the inflammatory cytokines (such as IL-1 and IL-6) can suppress PTH secretion and disturb vitamin D metabolism, thereby perpetuating a cycle of hypocalcemia [45,110]. This “butterfly effect” illustrates how a single inflammatory trigger can cascade through multiple pathways, ultimately reprogramming the body’s calcium regulatory set-points—a phenomenon that is invisible when focusing on isolated parameters.

Systems biology’s integrative perspective enables the identification of these latent drivers and reveals the adaptive, network-driven nature of the disease. It allows researchers to develop robust computational models and high-throughput diagnostic tools that target fundamental system imbalances rather than merely addressing the manifestation of hypocalcemia. This holistic approach is leading the way toward more durable, comprehensive treatment strategies that can mitigate repeated episodes and improve overall dairy herd health.

In summary, while reductionist approaches have historically provided important foundational knowledge, they fall short in addressing the full complexity of milk fever. By embracing the dynamic interplay and nonlinear interactions among various physiological networks, the systems biology paradigm shift captures the emergent properties of the disease. A cow’s immune, endocrine, and metabolic systems are interconnected, meaning even small perturbations can have far-reaching consequences. This integrated framework not only deepens our understanding of disease etiology but also sets the stage for the development of more effective and sustainable management strategies in veterinary medicine [64,118].

## 12. Conclusions and Future Directions

Milk fever exemplifies how the traditional single-nutrient deficiency paradigm has overshadowed the deeper immunometabolic complexities underlying the disease. Emerging evidence indicates that hypocalcemia is not an isolated disorder but rather the consequence of interconnected inflammatory loops, endocrine imbalances, and metabolic stressors, including endotoxin translocation and chronic low-grade inflammation. These findings highlight the necessity of moving beyond a calcium-centric approach toward addressing broader systemic dysfunctions contributing to both clinical and subclinical manifestations.

A systems biology perspective positions milk fever as an emergent outcome of complex interactions among genetic, metabolic, immunological, and environmental factors. Multi-omics approaches have identified key pathways associated with calcium homeostasis and immune function, revealing that inflammatory markers and metabolic shifts frequently precede clinical hypocalcemia. These insights challenge conventional understanding and call for integrative research to refine mechanistic explanations of the disease.

Future advances in milk fever management should prioritize predictive modeling and early detection by integrating multi-omics biomarkers with real-time physiological monitoring. Precision nutrition strategies must be designed to enhance metabolic and immune resilience throughout the periparturient period, incorporating functional nutrients, probiotics, and dietary interventions that modulate inflammation and optimize calcium regulation. Additionally, genetic selection efforts should focus on incorporating resilience markers into breeding programs to accelerate the development of cows inherently resistant to milk fever and related metabolic disorders.

Given the complexity of milk fever, an interdisciplinary approach involving veterinarians, nutritionists, geneticists, and data scientists is essential to translating advanced research into practical, on-farm solutions. Collaborative efforts will bridge the gap between laboratory discoveries and applied dairy management, ensuring that new strategies are both scientifically sound and economically viable.

The future of milk fever prevention and treatment lies in embracing a holistic, systems-based approach that integrates predictive modeling, precision nutrition, targeted genetic selection, and interdisciplinary collaboration. These strategies offer the potential to transform dairy herd management, improve animal health and welfare, and enhance overall farm productivity and sustainability.

The insights presented in this review challenge the traditional understanding of milk fever and highlight the need for a broader, integrative approach. However, certain elements of this proposed framework require further experimental validation to establish a more comprehensive explanation of the disease. Future research should focus on refining these concepts through mechanistic studies and multi-omics analyses, ultimately leading to more effective prevention and management strategies. Longitudinal studies tracking inflammatory and calcium-related biomarkers during the prepartum and postpartum periods, as well as controlled inflammatory challenges independent of colostrogenesis, are essential to elucidate causal relationships. Supporting this direction, a study conducted by our team [13] found that concentrations of serum IL-6, TNF-α, serum amyloid A (SAA), haptoglobin, and lactate were significantly elevated in cows with milk fever compared to healthy controls at multiple time points. Importantly, serum TNF-α, SAA, haptoglobin, and lactate were already increased at −8 and −4 weeks prior to parturition, suggesting that systemic inflammation may precede and contribute to the development of hypocalcemia. These findings underscore the need to further investigate inflammation as a potential initiating factor—“the chicken” rather than “the egg”—in the pathophysiology of milk fever.

## Data Availability

Not applicable.
